# Green space exposure and colorectal cancer: A systematic review

**DOI:** 10.1016/j.heliyon.2023.e15572

**Published:** 2023-04-19

**Authors:** Noor Azreen Masdor, Maryam Fatimah Abu Bakar, Rozita Hod, Azmawati Mohammed Nawi

**Affiliations:** Department of Public Health Medicine, Universiti Kebangsaan Malaysia, Kuala Lumpur, 56000, Malaysia

**Keywords:** Colorectal cancer, Green space, Incidence, Ecosystem

## Abstract

Green space has been linked to colorectal cancer, but the evidence is still limited and inconclusive. This review aimed to investigate the relationship between green space and CRC. The studies were searched using three primary journal databases: PubMed, Scopus, and Web of Science. The retrieved citations were screened, and data from articles about GS exposure and CRC were extracted. The Newcastle-Ottawa Quality Assessment Form for Cohort Studies was used to evaluate the studies' quality. Five of the 1792 articles identified were eligible for the final review, which included five cohort studies published between 2017 and 2022. Each one article from the United States, the United Kingdom, France, Belgium, and Germany and All studies are of high quality. Four studies reported CRC incidence and one study reported CRC mortality from GS exposure. There was no significant association between GS attributes (Normalized Difference Vegetation Index (NDVI), surrounding greenness, surrounding green area, proximity to GS (agricultural lands, urban GSs, and forests), and count of recreational facilities and parks) with CRC. Only one study discovered that a healthier ecosystem was linked to a lower CRC risk. Although the evidence is still limited, the findings may indicate the presence of other factors in the relationship between GS and CRC. Future research should continue to focus on the variation of GS and the factors that influence it. Specific attention to the development of GS has the potential to produce benefits while mitigating cancer risk.

## 1 Introduction

Colorectal cancer is the most common gastrointestinal malignancy diagnosed in the world, with an estimated number of new cases in 2020 for both sexes, about 1.9 million and 900,00 deaths [1]. Years of research have identified several environmental CRC risk factors, yet the precise causes of colorectal neoplasms remain unknown [2].

Rapid economic growth, increased exposure to environmental and lifestyle risks, associated with the increased incidence of CRC [3]. The development of CRC further increased with the combination of environmental and individual-level risk factors, such as lifestyle and habits related to diet, smoking and alcohol consumption [4]. Exposure to GS also affects public health, e.g. in terms of prevalence of chronic disease [5], mental health (depression symptoms) [6] and mortality [7].

Green spaces are often associated with nature and contain green vegetation [8]. They are also closely related to physical activity because they can encourage walking, running, and playing [9]. Convincing evidence suggests that physical activity (of all types and intensities) lowers the risk of colon cancer, but no conclusion has been reached for rectal cancer [4].

There has been a theory that physical activity affects proximal and distal colon cancers differently, but a systematic review and meta-analysis suggest that the risk of colon cancer does not differ by anatomical subset [10]. According to a meta-analysis, physical activity reduces the risk of colon cancer by 24% overall [11] and associated with a lower risk of death from colorectal cancer [12]. Sedentary lifestyles and physical inactivity are linked to an increased risk of CRC, according to researches [[13], [14], [15], [16]]. A meta-analysis demonstrated that occupational sitting and TV viewing time are associated with an increased risk of CRC [17]. Physical inactivity can lead to physiological changes such as prolonged intestinal transit time, insulin resistance and increased chronic inflammation, all of which are involved in the development of CRC [18]. CRC was also explained as a result of metabolic disruption, genomic instability, and oxidative stress in humans, all of which are linked to a sedentary lifestyle, obesity and overweight [3]. Mechanisms underlying the effect of green space exposure, availability, and accessibility on physical activity and body mass index are thought to have an impact on CRC risk.

Researchers are currently interested in residential environmental risk factors and their relationship to noncommunicable diseases [19,20] and cancer [21,22]. Many recent studies highlighted the environmental colorectal cancer risk factors. A systematic review and meta analyses on the relationship between exposure to greenspace and cancer incidence, prevalence, and mortality have been conducted [23]. However, there is surprisingly little research on a possible link between GS and its impacts on CRC.

The aim of this study was to investigate the association between exposure to GS in the adult population and outcomes of CRC (including incidence and mortality). The results may contribute to a better understanding of risk factors important for the development of effective environmental intervention strategies.

## 2 Materials and methods

This review is registered under PROSPERO (CRD42022374803).

This review followed the protocol established by Preferred Reporting Items for Systematic Reviews and Meta-Analyses (PRISMA) 2020 to search the relevant literature comprehensively. The review was guided by population, exposure, and outcome (PEO) to determine the relevant articles. Based on this concept, three significant aspects were included: adult participants with no gender or area restriction (population), GS (exposure) and CRC outcome (outcome), which were then combined to form the primary research question: What impact does the exposure of the GS environment (E) have on the CRC outcome (O) in the adult population (P)? [24].

### 2.1 Data source and search strategy

The relevant keywords were identified using Medical Subject Headings (MeSH), dictionaries and thesauri after discussing them with team members. During the search process, one team member (N.M.) used relevant keywords to search for potentially relevant studies from November 2022. Specific keywords were then developed using an advanced search with adjacency operators, truncation and Boolean operators. The keywords were used to search for articles in three databases: Web of Science (WOS) including WOS Core Collection, PubMed and Scopus, see Table S1 in the supplementary material.

### 2.2 Selection process

Records were identified in the databases. Duplicate articles were removed and given for screening. Screened articles were then selected based on inclusion and exclusion criteria. Accepted articles were primary original articles written in English by November 1, 2022 based on the following criteria.

(a) Population: adult participants with no gender or area restriction
(b) Exposure: Studies that assessed the quantity or quality of green spaces on all types of natural and man-made green environments such as parks, playgrounds, coastal parks with vegetation, etc. were included as long as they were defined as green spaces by the authors.
(c) Outcomes: Studies that examined CRC risk or outcomes, including prevalence, incidence and mortality
(d) Study design: All observational and intervention studies, including randomised, quasi-randomised and non-randomised studies.

We excluded articles that did not refer to CRC, GS environment and CRC outcome or studies with non-human subjects, study protocols, conference abstracts, dissertations, reviews, qualitative studies, editorials, case studies and opinion articles. In the selection of articles, all authors (N.M., A.N., M.A, R.H.) were involved in reviewing the titles and abstracts of potentially eligible articles. Each article was independently reviewed by at least two authors. Any disagreements were resolved by discussion and consensus between two authors or with the help of the research team leader. The full text was obtained and thoroughly reviewed to determine if it met the inclusion criteria and objectives of the review. Articles were rejected if they did not answer the research questions.

### 2.3 Data extraction and synthesis

The included studies were then downloaded and retrieved by the researchers (N.M., A.M.). Data from the completed studies, including authors' names, article title, year, country, study location, study population, sample size, study objectives and summary of results, were extracted by the authors and collected in an Excel file. The results were summarised in a narrative synthesis. Prior to data extraction and analysis, the authors assessed the methodological quality of the included studies using the Newcastle-Ottawa Quality Assessment.

### 2.4 Assessment of methodological bias

The authors utilized Newcastle-Ottawa Quality Assessment Form for Cohort Studies (NOS) [25] to assess the risk of bias and ensure the study's quality. The NOS came from an ongoing collaboration between the Universities of Newcastle, Australia and Ottawa, Canada. According to Ma et al. [26], among the many tools, NOS is the most commonly used, which can also be adapted to specific topics. This tool has three parts: selection (4 questions), comparability (1 question) and outcome (3 questions). The final scores will be divided into three categories: good (3 or 4 stars in the selection domain and 1 or 2 stars in the comparability domain and 2 or 3 stars in the outcome/exposure domain), fair (2 stars in the selection domain and 1 or 2 stars in comparability domain and 2 or 3 stars in outcome/exposure domain) and poor (0 or 1 star in selection domain or 0 stars in comparability domain or 0 or 1 stars in outcome/exposure domain). All included studies received one star for the questions in each section of the checklists. Articles were selected if both reviewers agreed on the quality of the articles. In case of disagreement, the assigned reviewers consulted a third independent reviewer. The quality scores are shown in Table 1.

**Table 1:** Detailed Newcastle-Ottawa Scale of each included cohort study.

Authors	Selection				Comparability		Outcome			Total score	Quality
	Represen-tativeness of exposed cohort	Selection of non-exposed cohort	Ascertainment of exposure	Demonstration that outcome of interest was not present at start of study	Adjust for the most important risk factors	Adjust for other risk factors	Assessment of outcome	Follow-up Length	Loss to follow-up rate		
Chen et al. [27]	1	0	1	1	1	1	1	1	0	7	Good
(Canchola et al., 2017)	1	1	1	1	1	1	1	1	1	9	Good
Sakhvidi et al. [28]	1	1	1	1	1	1	1	1	1	9	Good
Rodriguez-Loureiro et al. [29]	1	1	1	1	1	1	1	1	0	8	Good
Datzmann et al. [30]	1	1	1	1	1	1	1	1	0	8	Good

## 3 Result

**Fig. 1:**
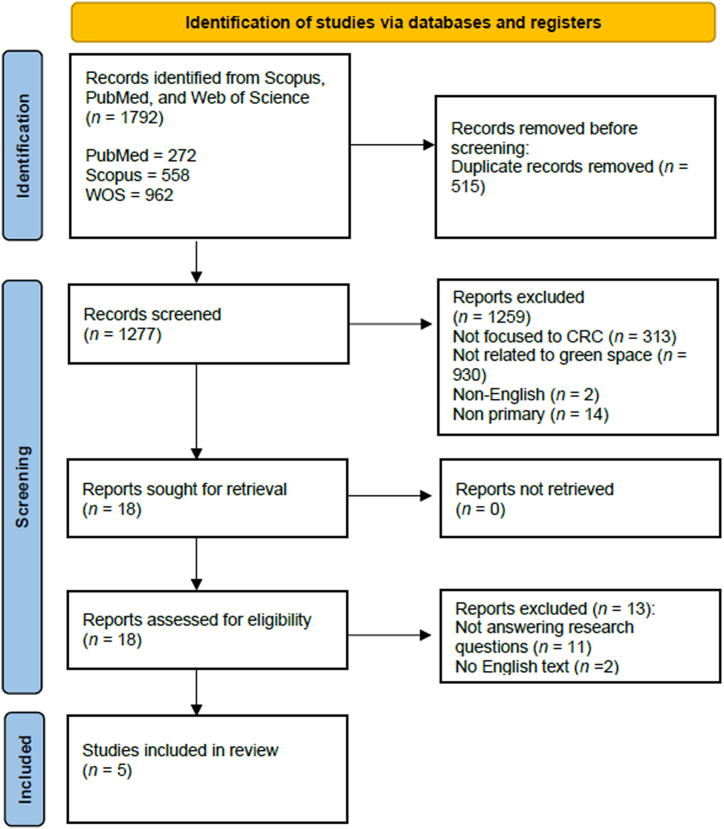
PRISMA flow diagram.

The search was then carried out according to the PRISMA flow, as shown in Fig. 1.

The initial search returned 1792 studies. 515 duplicate articles were removed and then the titles and abstracts of the remaining 1277 articles were reviewed to see if they answered the research questions. Of the 1277 articles, 18 were accepted and 1259 were excluded (313 were not focused on CRC, 930 were not related to green spaces, two were not in English, 14 were not primary. Thirteen articles were rejected because they did not answer the research questions, leaving a total of five articles.

Due to the considerable heterogeneity, the data are presented descriptively.

### 3.1 Characteristics of the included studies

This review covers five publications published between 2017 and 2022. All five articles were cohort studies, each from the United States [31], the United Kingdom [27], Germany [30], France [28] and Belgium [29], with high quality evaluated based on Newcastle-Ottawa Quality Assessment Form for Cohort Studies. The study population was aged between 30 and 69 years and the sample size ranged from 19408 to 2441566 participants. Recruitment took place from 1989 to 2010. The shortest follow-up period was five years and the longest was 27 years.

Table 2presents the characteristics of the included studies.

**Table 2:** Characteristics of included studies.

Authors	Country	Study design	Study population	Recruitment area	Sample size	Year of recruitment	Length of follow up (years)
Chen et al. [27]	UK	Cohort	1) Aged 40–69 years	England, Scotland, and Wales	335,370	2006–2010	8.69 (Median follow up)
Canchola et al. [31]	US	Cohort	1) Participants from Multiethnic Cohort that completed a baseline questionnaire	Hawaii and California	81,197	1993–2010.	16.6 (Median follow-up)
Sakhvidi et al. [28]	France	Cohort	1) Aged at enrolment in the range of 35–50	French national electricity and gas company worker (Électricité de France-Gaz de France) workers	19,408	1989	27
Rodriguez-Loureiro et al. [29]	Belgium	Cohort	1) Aged 30 years or older	Belgium (Antwerp, Ghent, Brussels, Charleroi, and Li'ege).	2,441,566	2001	13 (2001–2014)
Datzmann et al. [30]	German	Semi -individual cohort	AOK PLUS beneficiaries in Saxony	Saxony	1,918,449	2008 and 2009	5

### 3.2 GS attributes

Table 3presents the description and interpretation of the GSs. The decision to include attributes was based on the broad definition found in the articles by Taylor and Hochuli [17] and Markevych et al. [32].

**Table 3:** GS attributes in the included study, description and the exposure assessment details.

Authors	Data source	Attributes	Description	Buffer	Consider exposure time window?	Time dependent approach	Point of time measure	Address used	Movers/not considered
Chen et al. [27]	UKB	Garden percentage	the percentage of the home location buffer classed as ‘Domestic garden'	300 m	–	–	–	House address	–
	Land Cover Map (LCM) 2007	Natural environment percentage	the percentage of the home location buffer classed as ‘Natural Environment'		–	–	–		–
	Median garden percentage and the median natural environment percentage	Ecosystem count	To indicate better ecosystem		–	–	–		–
Canchola et al. [31]	California Neighborhoods Data System	Recreational facilities and parks count	Total number of recreational facilities and parks	1600 m	Difficult to capture critical exposure window	Done but no associations	One point, At baseline	Baseline residential addresses	No information
Sakhvidi et al. [28]	European CORINE land cover	Residential proximity to the different types of greenspace: agricultural lands, urban greenspace, and forests	Euclidean distance from each residential address to nearest distances	100, 300, 500, and 1000 m radius	Yes, 5–10 year delay between exposure and outcome	Providing sensitivity analyses on 5–10 years lag	1990, 2000, 2006, and 2012	Residential address	Yes - “resindential mobility”, categorized movers and non movers
	Remote sensing data of Landsat satellites	Residential surrounding greenness	the normalized difference vegetation index (NDVI)				used the NDVI values at the greenest time of each year		
Rodriguez-Loureiro et al. [29]	the Landsat 5	Surrounding greenness	NDVI	300, 500, 1000 m	Consider the exposure close to the middle of the follow-up period	–	summer period of 2006, with a 30 × 30 m resolution.	Residential address	Yes, Subgroup analysis included on who resided in the same census tract for 10 years
	Urban Atlas 2006 (UA)	Surrounding green areas	the percentage of surface around the residential address covered by green areas			–	Data on 2006 only		
Datzmann et al. [30]	MODerate-resolution Imaging Spectroradiometer (MODIS) satellite images at the resolution of 250 m	Surrounding greenness	NDVI	–	–	–	16-day omposite NDVI images for the years 2005–2009	Residential address	Yes

The attributes included in this review GS are the percentage of gardens and natural environment grouped as ecosystem [27], the recreational facilities and parks count [31], residential proximity to the various greenspaces, residential surrounding greenness [28], surrounding greenness using the Normalized Difference Vegetation Index (NDVI) [[28], [29], [30]] and surrounding green areas [29]. A further description of the attributes can be found in Table 3. Basically, all studies used the residential address as the reference point for measurement. To measure exposure, each study used different buffer sizes depending on the attributes. For example, the most common buffer size was 300 m used for the garden, and the natural percentage [[27], [28], [29]]. Sakhvidi et al. [28] have different buffers as they investigated different types of green areas: agricultural land, urban green areas and forests. One study [28] includes the data of the time window of exposure. A time-dependent approach was used by two studies [28,31] when looking for recreational facilites and parks. Four studies mentioned the specific time for measuring GS and CRC [[27], [28], [29], [30], [31]]. Three studies analysed the data of movers compared to non-movers and look for differences in the GS exposure.

### 3.3 CRC outcome

The CRC outcomes were categorized as the CRC incidence and mortality rate. Four studies reported GS exposure and CRC incidence [27,28,30,31], while one reported CRC mortality [29]. Only research from Canchola et al. [31] separated colon and rectal cancer while others studied on overall colorectal cancer. The findings are summarised in Table 4.

**Table 4:** CRC data source, number of cases. Code used and type of cancers identified in the included studies.

Authors	CRC outcome	CRC data source	Numbers of CRC cases	Codes for outcome	Type of cancer analyzed (overall CRC, colon or rectal cancer)
Chen et al. [27]	Incidence	UK Biobank (UKB) and coded using ICD-9 or ICD-10.	10,702	ICD-9153–1541, 2113, 2114, 2303, 2004, 1975, and 2352, or ICD-10 C180, C182–C189, C19, C20, D12–D129, D10, C785, and D374.	Overall CRC
Canchola et al. [31]	Incidence	1) CRC incidence cases from California Cancer Registry	1973	ICD-O-3 site codes C18.0-C18.9, C26.0, C19.9, and C20.9	Overall, separate analyses on colon and rectal cancer
		2) Mortality data from California death certificate files and the National Death Index.		The first diagnosis of invasive CRC (ICD for Oncology-3	
Sakhvidi et al. [28]	Incidence	1) Cancer records collected by the Électricité de France- Gaz de France department. 2) the cancer validation platform PRIMEV’R and SNIIRAM database (Système National d’Information Inter-Régimes del’Assurance Maladie)	4075	ICD-10 code: C18 for colon cancer; C19 for rectosigmoid cancer; C20 for rectum cancer	Overall CRC
Rodriguez-Loureiro et al. [29]	Mortality	Mortality data provided by death certificates	13,618	ICD-10 code: C18–C22	Overall CRC
Datzmann et al. [30]	Incidence	Aok PLUS data on beneficiaries diagnosed with cancer from 2010 to 2014	11,975	C18–C21	Overall CRC

### 3.4 The link between GS and CRC

#### 3.4.1 GS and CRC incidence

Table 5summarised the association between GS attributes with CRC. Only one study reported that a better ecosystem which includes the garden and natural environment, is associated with a lower risk of CRC [27]. Another study with a 27-year follow-up cohort suggested that greenspace has a protective role for colorectal cancers, but the findings were not statistically significant [28]. Similarly, Datzmann et al. [30] discovered no link between GS (NDVI) and CRC. Another study by Canchola et al. [31] found no significant associations between recreational facilities, park count, and colorectal cancer risk.

**Table 5:** The association between the GS attributes and CRC.

Authors	Statistical analyses	Environment	Comparator	CRC outcome						Interpretation & Conclusion
				Incidence	95% CI	p-value	Mortality	95% CI,	p-value	
Chen et al. [27]	^1^Factor analysis	Ecosystem		HR = 0.970	0.952,0.989	0.00				1) The ecosystem was positively correlated with a reduced risk of CRC 2) A decreased incidence of CRC ecosystem count 1 and 2. 3) A better ecosystem was associated with a reduced risk of CRC
		Garden percentage (300 m buffer)		HR = 0.996	0.994,0.998	0.00				
		Natural environment percentage (300 m buffer)		HR = 0.999	0.998,1.000	0.03				
		Ecosystem count (combined the median garden percentage and natural environment percentage)								
		Count 1	Count 0	HR = 0.931	0.879, 0.985	p < 0.05				
		Count 2	Count 0	HR = 0.910	0.847, 0.978	p < 0.05				
		No sensitivity analysis								
Canchola et al. [31]	^2^Multivariable Cox regression models. adjusted for the following individual-level CRC	Male (overall)								
		Number of recreational facilities								
		2	3+ (ref)	1.10	0.91–1.34	–				1) No significant associations between recretional facilities and parks count and colorectal cancer risk in male
		1		1.15	0.95–1.39	–				
		0		1.04	0.85–1.29	–				
		0 (White, male)		1.37	0.75–2.49	–				
		p-trend	0.45							
		Female (overall)								
		2	3+ (ref)	0.97	0.80–1.18	–				1) No significant associations between recretional facilities and parks count and colorectal cancer risk in female 2) No versus 3+ recreational facilities was associated with an increased CRC risk in White female
		1		1.02	0.85–1.24	–				
		0		0.92	0.75–1.12	–				
		0 (White, female)		1.64	1.02, 2.65	p < 0.05.				
		p-trend	0.82			–				
Zare Sakhvidi et al. [23]	^3^Time-dependent Cox proportional-hazard regression models	NDVI at 100 m	Urban	0.883	0.624, 1.250	–				Non-significant associations were observed
			Semi urban	0.938	0.692, 1.272	–				
			Rural	0.989	0.743, 1.315	–				
		Proximity to agricultural lands		0.994	0.829, 1.191	–				Proximity to different types of GS (agricultural lands, urban GSs, and forests) was not associated with increase or decrease in the CRC risk
		Proximity to forests		1.027	0.807, 1.307	–				
		Proximity to urban GSs	Urban	1.846	0.962, 3.541	–				
			Semi urban	0.983	0.863, 1.119	–				
	Extended Cox models	NDVI at 100 m		0.991	0.823, 1.194	–				Srrounding greenness not associated with a significant increase or decrease in the CRC risk
		NDVI at 300 m		1.002	0.813, 1.235	–				
		NDVI at 500 m		0.967	0.781, 1.197	–				
		NDVI at 1000 m		0.904	0.729, 1.122	–				
		Proximity to agricultural lands		0.977	0.905, 1.054	–				Proximity to different types of GS (agricultural lands, urban GSs, and forests) was not associated with a significant increase or decrease in the CRC risk
		Proximity to forests		1.049	0.936, 1.176	–				
		Proximity to urban GSs		0.945	0.853, 1.047	–				
		Sensitivity analyses: occupational exposure, with 5 years lag of 10-years moving average of exposure term on subjects without mobility, with no lag of 10-years moving average of exposure term, (with 10 years lag of 10-years moving average of exposure term) on subjects without mobility,								Non-significant associations were observed
Rodriguez-Loureiro et al. [29]	^4^Mixed-effects Cox proportional hazards models	Surrounding greenness					0.969	0.936, 1.003	–	No significant associations between exposure to GSs and colorectal cancer mortality after full adjustment.
		Surrounding green area					0.985	0.958, 1.013	–	
		Sensitivity analyses					Different buffer sizes for surrounding green spaces, limited our analyses to non-movers, residents with Belgian origin, a healthy subpopulation and individuals residing in the city			
		Non movers Surrounding greenness, 300-m					0.979	0.943, 1.017	–	No significant associations between exposure to GSs and colorectal cancer mortality
		Surrounding green areas, 300-m					0.990	0.961, 1.020	–	
		Perceived neighbourhood greenness					0.980	0.953, 1.008	–	
		Belgian origin, Surrounding greenness, 300-m					0.963	0.928, 0.999	–	
		Belgian, Surrounding green areas, 300-m					0.986	0.957, 1.015	–	
		Belgian, Perceived neighbourhood greenness					0.973	0.946, 1.000	–	
		Other variables show no significant results								
Datzmann et al. [30]	^5^Multilevel Poisson models with 95% confidence intervals with the R software package lme4	NDVI (10% increase)		RR = 1.03 R^2^ = 0.004	0.98, 1.07	–				No association was found between GS (NDVI) and CRC.
		Sensitivity analyses: male and alcohol related disorder								
		Male sex		RR = 1.78	1.71, 1.84	–				In sensitivity analyses, male and alcohol related disorder found increased risk for CRC with increasing 10% of NDVI, sex and alcohol potential cofounders
		Alcohol-related disorder		RR = 1.50	1.38,1.63	–				

#### 3.4.2 GS and CRC mortality

In their 13-year follow-up cohort study, Rodriguez-Loureiro did not find a significant connection between CRC mortality and GS (greenness and green areas) [29].

## 4 Discussion

This review investigated the association between GS and CRC. According to the literature, the term “GS” appears in many academic articles, including architecture, urban planning, building science, biology, medicine, and health [8]. The term “GS” also refers to parks, gardens, yards, urban forests, and urban farms are examples of urban vegetation. Landscape vegetation includes forests and wilderness areas, street trees and parks, gardens and backyards, geological formations, farmland, coastal areas, and food crops [8]. The GS is measured in various ways, such as using normalized difference vegetation index (NDVI), street view, tree density, greenspace percentage, and landscape percentage from remote sensing methods. WHO [33] suggested that the percentage of green space on land cover and land use maps, such as the European Urban Atlas, could be an important indicator.

Secondary indicators include satellite-based NDVI and perception-based measures of urban GS. Some studies have used green space density indicators in conjunction with national or local land use/land cover datasets. Other studies have used international data, such as Coordination of Information on the Environment (CORINE) land cover data. Each map has a minimum unit. The mean NDVI value for an area is calculated using high-resolution imagery and serves as an indicator of the “greenness” of the environment. At a certain distance from the focal point of a geographic region, the area can be considered a “buffer” zone”. These databases can be examined with different buffer sizes (100, 300, 500, 1000 and 3000 m) [34].

Because there is no universally accepted definition of “GS,” even standardisation is favourable; however, forcing “GS” to mean the same thing everywhere is detrimental; thus, the GS accepted for inclusion in this review are the ecosystem (because it includes a garden and natural environment), recreational facilities and parks within the residence, and residential proximity to various types of GS surrounding green area and surrounding greenness. As a result, this review provides a relevant definition of the term for each study.

For now, no evidence supports the association between GS and CRC. A study by Rodriguez-Loureiro in their 13 years follow-up cohort study reveals that residing in GS (greenness and green areas) could minimize the risk of mortality from lung and breast cancer but not CRC [29]. The study also stated that the lack of association could be attributed to a lack of statistical power due to the smaller sample size and shorter follow-up period. Another study with a 27-year follow-up cohort suggested that greenspace has a protective role for colorectal cancers, but the findings were not statistically significant [28]. Their study had some limitations, including the fact that it focused on a different type of greenspace, that exposure ratings varied greatly, and that the study lacked statistical power. Similarly, Datzmann et al. [30] revealed no link between GS (NDVI) and CRC while Canchola et al. [31] found no significant associations between recreational facilities, park count, and colorectal cancer risk.

A better ecosystem also means healthy and balanced ecosystem, which include the green and natural environment might provide a greener and less polluted environment. Only one study in this review found that a better ecosystem, including the garden and natural environment, was associated with a lower risk of CRC [27]. There has been little research comparing the ecosystem and CRC to other cancers. Natural and artificial GS are considered ecosystem services as they fulfill basic needs such as food, water, and air, as well as regulating air quality, vector-borne disease, climate change, and facilitating living, recreational, and spiritual interactions with nature to enhance human well-being [35,36]. Increased exposure to better ecosystem strengthens the immune system by altering the human microbiome, provides long-term health benefits, lowers the incidence of non-communicable diseases and reduces mortality [37].

GS research in CRC is still restricted compared to other cancer such as lung, breast, and prostate. A meta-analysis found no statistically significant links between greenspace and breast, lung, or prostate cancer incidence [23]. A population-based case-control study in Madrid shows an association between urban GS and the reduction of childhood leukaemia incidence [38]. In a study conducted in Spain urban GSs, including gardens, zoos, and urban parks, lowered breast cancer risk [39]. GS was associated with a lower incidence of lethal prostate cancer in a study conducted in the United States [21] but contrary results showed that GS exposure increases prostate cancer mortality [29].

Agricultural land provides greenery, urban attractiveness, and food production, but also an increased risk of cancer. In a study by Sakhvidi et al. [28] observed an increased risk of prostate, breast, colorectal (but not significant), bladder, lung, and malignant melanoma of the skin in proximity to agricultural lands. The results also show that proximity to agricultural areas increases the risk of breast cancer in Spain [39]. Agricultural land can also be classified as GS, but the benefits do not resemble those of parks [40]. It has been proposed that increased pesticide exposure is linked to cancer [41]. The evidence for the other cancers was inconclusive; therefore, further studies will have to consider the other types of greenspaces (for example agricultural area) and evaluate type of cancer [23].

Canchola et al. [31] found no significant associations between recreational facilities, number of parks and risk CRC. The number, availability and accessibility of parks and recreation facilities can influence on physical activity in adults [42] and children [43]. However, the Dutch National Health Survey, found that distance to the nearest GS is inconsistently associated with physical activity and obesity [34] which support for that not necessarily mean that person who live closest to a park are more likely to visit a park, exercise and have lower body weight compared to who live further away. This supports that according to one study, a long distance between children's homes and the nearest park was associated with a significantly lower risk of obesity in urban children [44].

Surrounding greenness may promotes positive behaviour like physical activity, social interaction and stress relief [45]. The normalized difference vegetation index (NDVI) is used as a indicator of surrounding greenness in this review. Included studies emphasised that CRC outcome was not affected by GS exposures because the influenced by individual and organizational components. GS may have lower road density and hence reduced traffic, resulting in lower levels of traffic-related air pollutants, whereas trees may restrict the dispersal of traffic air pollutants and thus increase the concentration of air pollution in the roads [32]. Apart of the availability of green areas, behaviour is also a main factor. The surrounding greenness may have no or little effect on people's levels of physical activity. Social Cognitive Theory states that people are driven by both internal and external forces [46]. This concept proposes that behaviour and environmental factors affect human action (physical activity). The socioecological framework (SEF) strengthens the theory that the component of the behavioural and organizational impacts on cancer prevention and control. This paradigm has been utilized to strengthen cancer prevention and control initiatives including in the Pacific Region [47].

Spatial analysis methods used to investigate the relationships between distance to green space and health data [48]. The general approach for calculating the GIS distance is the Euclidean distance and the network distance. The Euclidean distance in Sakhvidi et al. [28] is used to measure distance of each residential address (as a point) to the nearest, different type of GS. A straight-line distance or Euclidean distance is the length of a straight line connecting two target points. However, there are other factors that can alter the calculation of the straight-line distance, such as obstacles and surface distances. Nowadays, distance measurement has evolved from Euclidean distance to network-based distance [48]. The network distance is determined using network analysis techniques that calculate the shortest distance or travel time between two points in the network [49]. It is assumed that using network has better approximation to the real world. In this approach, the distance between two points is measured by the shortest path using network analysis. Compared to Euclidean distance, network distance is more accurate in estimating accessibility because residents use roads. However, to obtain more accurate data, network distance requires the creation of more detailed geospatial data in the software GIS. Using an inappropriate method to examine distance and access to green space can directly influence the direction (and extent) of the association and therefore limit its relevance in broader geographical contexts [48].

The research into GS was motivated by the positive health outcomes by acting as biological, chemical, physiological, psychological, and environmental agents to improve health and reduce disease risks [50]. Studies have linked greenspace and cardiovascular disease (CVD) outcomes, such as lower CV mortality, inspiring researchers to look for similar results in CRC [51]. GS can help to improvise the inflammatory profile through many interventions such as forest bathing inspired by traditional Japanese nature immersion to reduces stress and inflammation [52]. There is also the different profile of stress, antioxidant and cytokine between urban and forest environment exposure that can be used to connect it with CRC [53]. GS strategies such as urban planning involving GS could consider working with an interdisciplinary team of environmental specialists, public health professionals, epidemiologists, anthropologists and psychologists [54]. Local stakeholders and community organizations can participate in GS initiatives by identifying appropriate locations and initiatives. Creating new or modifying GS must be tailored to the needs of the public, particularly in disadvantaged communities. City planners play an important role in preventing the development of GSs. The best agricultural land and natural assets like parks, lakes, and riverfronts must be protected especially in highly populated places. Integrated city development incorporates ecological, environmental, and social justice considerations into land investment and development decisions [55]. Urban planning initiatives that protect GS align with promoting “sustainable cities” [56].

### 4.1 Challenges related CRC and GS research

Greenspace is directly and indirectly connected to other risk variables such as sun exposure, physical activities, obesity, and air quality, making it challenging to be evaluated [57]. The CRC investigations also need to consider the site of cancer, the type of greenspace, the measure of exposure, and the study's geographical location. The varying GS measurements utilized in that research may have led to conflicting findings [44]. The exposure to GS reduced risk of CRC in at least three ways, by decreases air pollutant concentrations [58], encouragement for walking and physical activities [59] and potential reduction in stress (the risk of rectal cancer but not colon cancer) [60]. However, the studies only examined exposure GS and not actual benefits such as reduction in pollutant concentration, increase in physical activity or stress reduction. None of the included studies examined the temporal relationship between exposure GS and outcome CRC (incidence and mortality), so the exact mechanism leading to diagnosis/death CRC or the possibility that CRC occurred several years/decades before diagnosis could not be investigated in this area. Lack of accuracy in reporting information may occur due to self-reporting [39]. The lack of association could be explained the other factors such as social background, safety, weather, mental health, and stress [39].

Socioeconomic background can have an impact on GS. Individuals with higher incomes and education levels have a greater opportunity to benefit from GS for their health than those in lower-income groups [61]. Exposure to varying levels of GS, duration, health status, and stress levels can all result in different outcomes. The study population may impact the results, so further research and sub-analysis may reveal some associations.

It is difficult to truly quantify the impact of GS because there are no specific indicators, as opposed to diet, where food directly affects human biological function. The pathway involved in the CRC mechanism is difficult to determine without using specific biomarkers and measuring the level of exposure to greenness. The mechanism of CRC carcinogenesis caused by GS is indirect and unclear. GS may have little influence on carcinogenesis through exposure to pollution [30], stress [22], physical activities [21] and obesity [62]. Molecular carcinogenesis research has advanced significantly, and scientists have uncovered numerous carcinogenic events. The interaction of genes and their environments revealed that genetic or epigenetic modifications displayed later in life may be influenced by what is exposed today.

### 4.2 Limitation and strength

Despite the higher quality of the available cohort studies on the association between GS and cancer, all studies found heterogeneous results for different subgroups. The main limitations that make it difficult to compare the results are that the association we observed is heterogeneous within and between studies, which is due to the studies having different exposure ratings, confounders and the power of the study in which the studies were involved. We found only five cohort studies on the association between green space and CRC, possibly due to the fact that the study relied on three databases, which might limit the number of relevant studies. Accepting articles written in English only could limit the number of potential native language studies to avoid additional costs, time and interpretation errors.

Many cohort studies contributed to identifying various GS-related risk factors associated with CRC incidence or mortality at the population level. Further randomized controlled trials that involve a large population may be required to validate our findings. With the availability of advanced technologies and data analysis, GS research has become an appealing method of discovering and monitoring environmental links with cancer trends.

## 5 Conclusions

This review addressed the current state of knowledge regarding the relationship between GS and CRC. According to the findings, there is currently no link between CRC and GS. GS is critical in promoting physical activity. Proximity to GS provides health benefits and reduces the risk of certain diseases, allowing those with access to choose the best disease prevention practice. As a result, comprehensive city planning is required to create a future environment that reduces CRC incidence and mortality. Validation of these findings in other studies and settings to further explore and advance our understanding of the potential health benefits of GSs.

## Author contribution statement

Noor Azreen Masdor, Azmawati Mohammed Nawi, Rozita Hod: Conceived and designed the experiments; Performed the experiments; Analyzed and interpreted the data; Contributed reagents, materials, analysis tools or data; Wrote the paper. Maryam Fatimah Abu bakar: Analyzed and interpreted the data; Wrote the paper.

## Data availability statement

No data was used for the research described in the article.

## Appendix A Supplementary data

Supplement table S2 Keywords_green space 2.0 Supplement table S2 Keywords_green space 2.0

## References

[1] Bray F., Ferlay J., Soerjomataram I., Siegel R.L., Torre L.A., Jemal A. (2018). Global cancer statistics 2018: GLOBOCAN estimates of incidence and mortality worldwide for 36 cancers in 185 countries. CA A Cancer J. Clin..

[2] Lewandowska A., Rudzki G., Lewandowski T., Stryjkowska-Góra A., Rudzki S. (2022). Title: risk factors for the diagnosis of colorectal cancer. Cancer Control.

[3] Rawla P., Sunkara T., Barsouk A. (2019). Epidemiology of colorectal cancer: incidence, mortality, survival, and risk factors. Przegląd Gastroenterol..

[4] World Cancer Research Fund/American Institute for Cancer Research (2018).

[5] Astell-Burt T., Feng X.Q. (2020). Urban green space, tree canopy and prevention of cardiometabolic diseases: a multilevel longitudinal study of 46 786 Australians. Int. J. Epidemiol..

[6] Patel D.M., Block R.C., Chapman B.P., Korfmacher K.S., van Wijngaarden E. (2019). Green space and mental health symptoms in a cardiac rehabilitation population. Indoor Built Environ..

[7] Villeneuve P.J., Jerrett M., Su J.G., Burnett R.T., Chen H., Wheeler A.J., Goldberg M.S. (2012). A cohort study relating urban green space with mortality in Ontario, Canada. Environ. Res..

[8] Taylor L., Hochuli D.F. (2017). Defining greenspace: multiple uses across multiple disciplines. Landsc. Urban Plann..

[9] Hankey S., Marshall J.D. (2017). Current Environmental Health Reports.

[10] Boyle T., Keegel T., Bull F., Heyworth J., Fritschi L. (2012). Physical activity and risks of proximal and distal colon cancers: a systematic review and meta-analysis. J. Natl. Cancer Inst..

[11] Wolin K.Y., Yan Y., Colditz G.A., Lee I.-M. (2009). Physical activity and colon cancer prevention: a meta-analysis. Br. J. Cancer.

[12] Cabilan C.J., Hines S. (2017). The short-term impact of colorectal cancer treatment on physical activity, functional status and quality of life: a systematic review. JBI Database of Systematic Reviews and Implementation Reports.

[13] An S., Park S. (2022). Association of physical activity and sedentary behavior with the risk of colorectal cancer. J. Kor. Med. Sci..

[14] Berkovic M.C., Cigrovski V., Bilic-Curcic I., Mrzljak A. (2020). What is the gut feeling telling us about physical activity in colorectal carcinogenesis?. World Journal of Clinical Cases.

[15] Cho Y.A., Lee J., Oh J.H., Chang H.J., Sohn D.K., Shin A., Kim J. (2019). Genetic risk score, combined lifestyle factors and risk of colorectal cancer. Cancer Res Treat.

[16] Papadimitriou N., Dimou N., Tsilidis K.K., Banbury B., Martin R.M., Lewis S.J., Kazmi N., Robinson T.M., Albanes D., Aleksandrova K., Berndt S.I., Timothy Bishop D., Brenner H., Buchanan D.D., Bueno-de-Mesquita B., Campbell P.T., Castellví-Bel S., Chan A.T., Chang-Claude J., Ellingjord-Dale M., Figueiredo J.C., Gallinger S.J., Giles G.G., Giovannucci E., Gruber S.B., Gsur A., Hampe J., Hampel H., Harlid S., Harrison T.A., Hoffmeister M., Hopper J.L., Hsu L., María Huerta J., Huyghe J.R., Jenkins M.A., Keku T.O., Kühn T., La Vecchia C., Le Marchand L., Li C.I., Li L., Lindblom A., Lindor N.M., Lynch B., Markowitz S.D., Masala G., May A.M., Milne R., Monninkhof E., Moreno L., Moreno V., Newcomb P.A., Offit K., Perduca V., Pharoah P.D.P., Platz E.A., Potter J.D., Rennert G., Riboli E., Sánchez M.J., Schmit S.L., Schoen R.E., Severi G., Sieri S., Slattery M.L., Song M., Tangen C.M., Thibodeau S.N., Travis R.C., Trichopoulou A., Ulrich C.M., van Duijnhoven F.J.B., Van Guelpen B., Vodicka P., White E., Wolk A., Woods M.O., Wu A.H., Peters U., Gunter M.J., Murphy N. (2020). Physical activity and risks of breast and colorectal cancer: a Mendelian randomisation analysis. Nat. Commun..

[17] Schmid D., Leitzmann M.F. (2014). Television viewing and time spent sedentary in relation to cancer risk: a meta-analysis. J. Natl. Cancer Inst..

[18] Chang W.Y., Chiu H.M. (2022). Beyond colonoscopy: physical activity as a viable adjunct to prevent colorectal cancer. Dig. Endosc..

[19] Rojas-Rueda D., Morales-Zamora E., Alsufyani W.A., Herbst C.H., AlBalawi S.M., Alsukait R., Alomran M. (2021). Environmental risk factors and health: an umbrella review of meta-analyses. Int. J. Environ. Res. Publ. Health.

[20] Yang M., Cheng H., Shen C., Liu J., Zhang H., Cao J., Ding R. (2020). Effects of long-term exposure to air pollution on the incidence of type 2 diabetes mellitus: a meta-analysis of cohort studies. Environ. Sci. Pollut. Control Ser..

[21] Iyer H.S., James P., Valeri L., Hart J.E., Pernar C.H., Mucci L.A., Holmes M.D., Laden F., Rebbeck T.R. (2020). The association between neighborhood greenness and incidence of lethal prostate cancer: a prospective cohort study. Environmental Epidemiology.

[22] Kondo M.C., Fluehr J.M., McKeon T., Branas C.C. (2018). Urban green space and its impact on human health. Int. J. Environ. Res. Publ. Health.

[23] Zare Sakhvidi M.J., Yang J., Mehrparvar A.H., Dzhambov A.M., Ebrahimi A.A., Dadvand P., Jacquemin B. (2022). Exposure to greenspace and cancer incidence, prevalence, and mortality: a systematic review and meta-analyses. Sci. Total Environ..

[24] Munn Z., MclinSc S.M., Lisy K., Riitano D., Tufanaru C. (2015). Methodological guidance for systematic reviews of observational epidemiological studies reporting prevalence and cumulative incidence data. Int. J. Evid. Base. Healthc..

[25] Wells G.A., Wells G., Shea B., Shea B., O'Connell D., Peterson J., Welch Losos M., Tugwell P., Ga S.W., Zello G.A., Petersen J.A. (2014).

[26] Ma L.-L., Wang Y.-Y., Yang Z.-H., Huang D., Weng H., Zeng X.-T. (2020). Methodological quality (risk of bias) assessment tools for primary and secondary medical studies: what are they and which is better?. Military Medical Research.

[27] Chen N., Liang H., Huang T., Huang N. (2022). Exposome approach for identifying modifiable factors for the prevention of colorectal cancer. Sci. Rep..

[28] Sakhvidi M.J.Z., Yang J., Siemiatycki J., Dadvand P., de Hoogh K., Vienneau D., Goldberg M., Zins M., Lequy E., Jacquemin B. (2021). Greenspace exposure and cancer incidence: a 27-year follow-up of the French GAZEL cohort. Sci. Total Environ..

[29] Rodriguez-Loureiro L., Verdoodt F., Lefebvre W., Vanpoucke C., Casas L., Gadeyne S. (2022). Long-term exposure to residential green spaces and site-specific cancer mortality in urban Belgium: a 13-year follow-up cohort study. Environ. Int..

[30] Datzmann T., Markevych I., Trautmann F., Heinrich J., Schmitt J., Tesch F. (2018). Outdoor air pollution, green space, and cancer incidence in Saxony: a semi-individual cohort study. BMC Publ. Health.

[31] Canchola A.J., Shariff-Marco S., Yang J., Albright C., Hertz A., Park S.Y., Shvetsov Y.B., Monroe K.R., Le Marchand L., Gomez S.L., Wilkens L.R., Cheng I. (2017). Association between the neighborhood obesogenic environment and colorectal cancer risk in the Multiethnic Cohort. Cancer Epidemiology.

[32] Markevych I., Schoierer J., Hartig T., Chudnovsky A., Hystad P., Dzhambov A., de Vries S., Triguero-Mas M., Brauer M., Nieuwenhuijsen M., Lupp G., Richardson E., Astell-Burt T., Dimitrova D., Feng X., Sadeh M., Standl M., Fuertes E. (2017). Exploring pathways linking greenspace to health: theoretical and methodological guidance. Environ. Res..

[33] WHO Regional Office for Europe (2016).

[34] Klompmaker J.O., Hoek G., Bloemsma L.D., Gehring U., Strak M., Wijga A.H., van den Brink C., Brunekreef B., Lebret E., Janssen N.A.H. (2018). Green space definition affects associations of green space with overweight and physical activity. Environ. Res..

[35] Aerts R., Honnay O., Van Nieuwenhuyse A. (2018). Biodiversity and human health: mechanisms and evidence of the positive health effects of diversity in nature and green spaces. Br. Med. Bull..

[36] van den Bosch M., Ode Sang Å. (2017). Urban natural environments as nature-based solutions for improved public health – a systematic review of reviews. Environ. Res..

[37] Rook G.A. (2013). Regulation of the immune system by biodiversity from the natural environment: an ecosystem service essential to health. Proc. Natl. Acad. Sci. USA.

[38] Ojeda Sánchez C., Segú-Tell J., Gomez-Barroso D., Pardo Romaguera E., Ortega-García J.A., Ramis R. (2021). Urban green spaces and childhood leukemia incidence: a population-based case-control study in Madrid. Environ. Res..

[39] O'Callaghan-Gordo C., Kogevinas M., Cirach M., Castaño-Vinyals G., Aragonés N., Delfrade J., Fernández-Villa T., Amiano P., Dierssen-Sotos T., Tardon A., Capelo R., Peiró-Perez R., Moreno V., Roca-Barceló A., Perez-Gomez B., Vidan J., Molina A.J., Oribe M., Gràcia-Lavedan E., Espinosa A., Valentin A., Pollán M., Nieuwenhuijsen M.J. (2018). Residential proximity to green spaces and breast cancer risk: the multicase-control study in Spain (MCC-Spain). Int. J. Hyg Environ. Health.

[40] Contesse M., van Vliet B.J.M., Lenhart J. (2018). Is urban agriculture urban green space? A comparison of policy arrangements for urban green space and urban agriculture in Santiago de Chile. Land Use Pol..

[41] Deziel N.C., Friesen M.C., Hoppin J.A., Hines C.J., Thomas K., Freeman L.E.B. (2015). A review of nonoccupational pathways for pesticide exposure in women living in agricultural areas. Environ. Health Perspect..

[42] Xiao Y., Miao S., Zhang Y., Xie B., Wu W. (2022). Exploring the associations between neighborhood greenness and level of physical activity of older adults in shanghai. J. Transport Health.

[43] Lachowycz K., Jones A.P., Page A.S., Wheeler B.W., Cooper A.R. (2012). What can global positioning systems tell us about the contribution of different types of urban greenspace to children's physical activity?. Health Place.

[44] Bloemsma L.D., Wijga A.H., Klompmaker J.O., Janssen N.A.H., Smit H.A., Koppelman G.H., Brunekreef B., Lebret E., Hoek G., Gehring U. (2019). The associations of air pollution, traffic noise and green space with overweight throughout childhood: the PIAMA birth cohort study. Environ. Res..

[45] James P., Banay R.F., Hart J.E., Laden F. (2015). A review of the health benefits of greenness. Current Epidemiology Reports.

[46] Marmo J. (2013). Applying social cognitive theory to develop targeted messages: college students and physical activity. West. J. Commun..

[47] Hurd T., Bartlett G., Nevarez L., Palafox N.A., Reichhardt M., Taitano J.R., Nitta M., Garstang H., Riklon S., Taulung L., Buenconsejo-Lum L.E. (2018). A socio-ecological framework for cancer control in the pacific: a community case study of the us affiliated pacific island jurisdictions 1997–2017. Front. Public Health.

[48] Higgs G., Fry R., Langford M. (2012). Investigating the implications of using alternative GIS-based techniques to measure accessibility to green space. Environ. Plann. Plann. Des..

[49] Cubukcu K.M., Hatcha T. (2016). Are euclidean distance and network distance related. Environment-Behaviour Proceedings Journal.

[50] Kuo M. (2015). How might contact with nature promote human health? Promising mechanisms and a possible central pathway. Front. Psychol..

[51] Bikomeye J.C., Beyer A.M., Kwarteng J.L., Beyer K.M.M. (2022). Greenspace, inflammation, cardiovascular health, and cancer: a review and conceptual framework for greenspace in cardio-oncology research. Int. J. Environ. Res. Publ. Health.

[52] Miyazaki Y. (2018).

[53] Im S.G., Choi H., Jeon Y.-H., Song M.-K., Kim W., Woo J.-M. (2016). Comparison of effect of two-hour exposure to forest and urban environments on cytokine, anti-oxidant, and stress levels in young adults. Int. J. Environ. Res. Publ. Health.

[54] Kondo M.C., South E.C., Branas C.C. (2015). Nature-based strategies for improving urban health and safety. J. Urban Health.

[55] Brown A. (2015).

[56] Vardoulakis S., Kinney P. (2019). Grand challenges in sustainable cities and health. Frontiers in Sustainable Cities.

[57] Wray A.J.D., Minaker L.M. (2019). Is cancer prevention influenced by the built environment? A multidisciplinary scoping review. Cancer.

[58] Pritchett N., Spangler E.C., Gray G.M., Livinski A.A., Sampson J.N., Dawsey S.M., Jones R.R. (2022). Exposure to outdoor particulate matter air pollution and risk of gastrointestinal cancers in adults: a systematic review and meta-analysis of epidemiologic evidence. Environ. Health Perspect..

[59] Morris J.S., Bradbury K.E., Cross A.J., Gunter M.J., Murphy N. (2018). Physical activity, sedentary behaviour and colorectal cancer risk in the UK Biobank. Br. J. Cancer.

[60] Kikuchi N., Nishiyama T., Sawada T., Wang C., Lin Y., Watanabe Y., Kikuchi S. (2017). Perceived stress and colorectal cancer incidence: the Japan collaborative cohort study. Sci. Rep..

[61] Crouse D., Pinault L., Balram A., Hystad P., Peters P., Chen H., Donkelaar A., Martin R., Ménard R., Robichaud A., Villeneuve P. (2017). Urban greenness and mortality in Canada's largest cities: a national cohort study. Lancet Planet. Health.

[62] Sanders T., Feng X., Fahey P.P., Lonsdale C., Astell-Burt T. (2015). Greener neighbourhoods, slimmer children? Evidence from 4423 participants aged 6 to 13 years in the Longitudinal Study of Australian children. Int. J. Obes..

