# Supplementary material for: Green space exposure and colorectal cancer: A systematic review
Source: Heliyon. 2023 Apr 19;9(5):e15572. doi: 10.1016/j.heliyon.2023.e15572 (PMC10160744; doi:10.1016/j.heliyon.2023.e15572)
Supplement: Supplement table S2 Keywords_green space 2.0 [file mmc1.docx]

Table S2. The keywords used to search for articles in three databases: Web of Science (WOS) including WOS Core Collection, PubMed and Scopus

| Search engine | Search area | Search date | Keywords | Number of articles |
| --- | --- | --- | --- | --- |
| Scopus | TITLE-ABS-KEY | From 12 -30 November 2022 | ( TITLE-ABS-KEY ( "Green space*" OR "greenspace*" OR "green?ess" OR "green infrastructure" OR "wild land" OR "natural land" OR "community land" OR "public land" OR "open land" OR "wild space" OR "natural space" OR "municipal park" OR "city park" OR "botanic park" OR "park access" OR "wild area" OR "natural area*" OR "green AND area" OR "woodland" OR "shinrinyoku" OR "forest AND bathing" OR "tree AND canopy" OR "garden*" OR "parkland*" OR "horticultur*" OR "forest*" OR "arboretum" OR "allotment" OR "natural AND facility*" OR "vegetation" OR "recreation" OR "green AND trail" OR "cycl* AND trail" OR "walk AND trail" OR "open AND space" OR "urban AND parks AND soils" OR "park*" OR "playground*" OR "beaches" OR "field*" OR "garden" OR "roadside AND green AND space" OR "residential AND green AND space" OR "attached AND green AND space" OR "built AND environment" OR "walkable" OR "walkability " OR "street AND connectivity" OR "land AND use" OR "residential AND density" OR "population AND density" OR "environment planning" OR "street AND planning" OR "neighborhood" OR "home AND environment" OR "urban AND design" OR "environment AND design" OR "residence AND characteristics" OR "geographic AND information AND sys*" OR "geographic AND mapping" OR "neighbourhood AND context" OR "walking AND locations" OR "street AND layout" OR "street AND design" OR "urban AND design" OR "urban AND planning" ) AND TITLE-ABS-KEY ( "Association" OR "Relation*" OR "Link" OR "connection" OR "association" OR "correlate" OR "tie" ) AND TITLE-ABS-KEY ( "risk" OR "possibility" OR "probability" OR "frequency" OR "predictor" OR "incidence" OR "occurrence" OR "rate" OR "prevalence" OR "mortality" OR "death" ) AND TITLE-ABS-KEY ( "Colorectal cancer" OR "colorectal neoplasm*" OR "colorectal carcinoma" OR "colorectal tumo*" OR "cancer colorectal" OR "bowel cancer" OR "large intestine cancer" ) ) | 962 |
| Pubmed | Title/Abstract | From 12 -30 November 2022 | ("green space*"[Title/Abstract] OR "greenspace*"[Title/Abstract] OR "green infrastructure"[Title/Abstract] OR "wild land"[Title/Abstract] OR "natural land"[Title/Abstract] OR "community land"[Title/Abstract] OR "public land"[Title/Abstract] OR "open land"[Title/Abstract] OR "natural space"[Title/Abstract] OR "municipal park"[Title/Abstract] OR "city park"[Title/Abstract] OR "botanic park"[Title/Abstract] OR "park access"[Title/Abstract] OR "wild area"[Title/Abstract] OR "natural area*"[Title/Abstract] OR "woodland"[Title/Abstract] OR "shinrinyoku"[Title/Abstract] OR "garden*"[Title/Abstract] OR "parkland*"[Title/Abstract] OR "horticultur*"[Title/Abstract] OR "forest*"[Title/Abstract] OR "arboretum"[Title/Abstract] OR "allotment"[Title/Abstract] OR "vegetation"[Title/Abstract] OR "recreation"[Title/Abstract] OR "park*"[Title/Abstract] OR "playground*"[Title/Abstract] OR "beaches"[Title/Abstract] OR "field*"[Title/Abstract] OR "garden"[Title/Abstract] OR "walkable"[Title/Abstract] OR "walkability"[Title/Abstract] OR "environment planning"[Title/Abstract] OR "neighborhood"[Title/Abstract]) AND ("Association"[Title/Abstract] OR "relation*"[Title/Abstract] OR "Link"[Title/Abstract] OR "connection"[Title/Abstract] OR "Association"[Title/Abstract] OR "correlate"[Title/Abstract] OR "tie"[Title/Abstract]) AND ("risk"[Title/Abstract] OR "possibility"[Title/Abstract] OR "probability"[Title/Abstract] OR "frequency"[Title/Abstract] OR "predictor"[Title/Abstract] OR "incidence"[Title/Abstract] OR "occurrence"[Title/Abstract] OR "rate"[Title/Abstract] OR "prevalence"[Title/Abstract] OR "mortality"[Title/Abstract] OR "death"[Title/Abstract]) AND ("Colorectal cancer"[Title/Abstract] OR "colorectal neoplasm*"[Title/Abstract] OR "colorectal carcinoma"[Title/Abstract] OR "colorectal tumo*"[Title/Abstract] OR "cancer colorectal"[Title/Abstract] OR "bowel cancer"[Title/Abstract] OR "large intestine cancer"[Title/Abstract]) | 272 |
| Web of Science | Topic | From 12 -30 November 2022 | "Green space*" OR "greenspace*" OR "green?ess" OR "green infrastructure" OR "wild land" OR "natural land" OR "community land" OR "public land" OR "open land" OR "wild space" OR "natural space" OR "municipal park" OR "city park" OR "botanic park" OR "park access" OR "wild area" OR "natural area*" OR "green AND area" OR "woodland" OR "shinrinyoku" OR "forest AND bathing" OR "tree AND canopy" OR "garden*" OR "parkland*" OR "horticultur*" OR "forest*" OR "arboretum" OR "allotment" OR "natural AND facility*" OR "vegetation" OR "recreation" OR "green AND trail" OR "cycl* AND trail" OR “walk AND trail" OR "open AND space" OR "urban AND parks AND soils" OR "park*" OR "playground*" OR "beaches" OR "field*" OR "garden" OR "roadside AND green AND space" OR "residential AND green AND space" OR "attached AND green AND space" OR "built AND environment" OR "walkable" OR "walkability " OR "street AND connectivity" OR "land AND use" OR "residential AND density" OR "population AND density" OR "environment planning" OR "street AND planning" OR "neighborhood" OR "home AND environment" OR "urban AND design" OR "environment AND design" OR "residence AND characteristics" OR "geographic AND information AND sys*" or "geographic AND mapping" or "neighbourhood AND context" or "walking AND locations" or "street AND layout" OR "street AND design" OR "urban AND design" OR "urban AND planning” (Topic) and “Association” OR “Relation*” OR “Link” OR “connection” OR “association” OR “correlate” OR “tie” (Topic) and “risk” OR “possibility” OR “probability” OR “frequency” OR “predictor” OR “incidence” OR “occurrence” OR “rate” OR “prevalence” OR “mortality” OR “death” (Topic) and “Colorectal cancer” OR “colorectal neoplasm*” OR “colorectal carcinoma” OR “colorectal tumo*” OR “cancer colorectal” OR “bowel cancer” OR “large intestine cancer” (Topic) | 558 |
